# Detection and persistence of environmental DNA from an invasive, terrestrial mammal

**DOI:** 10.1002/ece3.3698

**Published:** 2017-12-03

**Authors:** Kelly E. Williams, Kathryn P. Huyvaert, Kurt C. Vercauteren, Amy J. Davis, Antoinette J. Piaggio

**Affiliations:** ^1^ Wildlife Genetics Lab USDA, Wildlife Services, National Wildlife Research Center Fort Collins CO USA; ^2^ Department of Fish, Wildlife, and Conservation Biology Colorado State University Fort Collins CO USA; ^3^ School of Environmental and Forest Sciences University of Washington Seattle WA USA

**Keywords:** environmental DNA, invasive species, *Sus scrofa*, wild pig

## Abstract

Invasive *Sus scrofa*, a species commonly referred to as wild pig or feral swine, is a destructive invasive species with a rapidly expanding distribution across the United States. We used artificial wallows and small waterers to determine the minimum amount of time needed for pig eDNA to accumulate in the water source to a detectable level. We removed water from the artificial wallows and tested eDNA detection over the course of 2 weeks to understand eDNA persistence. We show that our method is sensitive enough to detect very low quantities of eDNA shed by a terrestrial mammal that has limited interaction with water. Our experiments suggest that the number of individuals shedding into a water system can affect persistence of eDNA. Use of an eDNA detection technique can benefit management efforts by providing a sensitive method for finding even small numbers of individuals that may be elusive using other methods.

## 1 INTRODUCTION

Biotic invaders are species that migrate, or are transported, to a new habitat in which subsequent generations reproduce, spread, and persist in the environment, often causing a negative impact on the newly colonized environment and biota (Mack et al., 2000). Life history traits such as high reproductive rate and rapid growth are core characteristics of successful, resilient invaders (Blackburn, Cassey, & Lockwood, 2009; Duncan, Blackburn, & Veltman, 1999; Sakai et al., 2001), but rapid dispersal by humans is also beneficial (Jeschke & Strayer, 2006).

Wild pigs have inhabited continental United States since the early 1500s after being introduced to Florida as domesticated European pigs (Mayer & Brisbin, 1991; Towne & Wentworth, 1950). Pigs were an important source of food for American settlers due to their adaptability and ability to survive in diverse habitats (Towne & Wentworth, 1950; West, Cooper, & Armstrong, 2009). Genetic data from various locations across Eurasia revealed multiple centers of independent domestication of wild pigs (Larson et al., 2005). Mitochondrial DNA sequences of pigs from the United States suggest a strong association between introduced pigs and European domestic breeds, thus reflecting the known history of human colonization and settlement of the United States (McCann et al., 2014). After the introduction of domesticated pigs, free‐range livestock management practices and escapes or release from enclosures led to the establishment of wild, or feral, pig populations across the country (Taylor, 1993). Hunting interests have prompted translocation of pigs throughout the United States, further contributing to rapid range expansion (McCann et al., 2014; Taylor, 1993). Aside from human‐assisted movements, characteristics of wild pigs that have made them a successful invasive species include high reproductive rates (Taylor, Hellgren, Gabor, & Ilse, 1998; Waithman et al., 1999) and that they are opportunistic generalists (Fogarty, 2007; West et al., 2009). Wild pigs can inhabit a multitude of habitat types, including harsh, seemingly uninhabitable regions such as deserts and northern latitudes with long winters (Adkins & Harveson, 2007; Corn et al. 2017; West et al., 2009; Wyckoff, Scott, Tyler, David, & Kurt, 2012), and due to their adaptability, suitable habitats occur throughout most of the country (Bevins, Pedersen, Lutman, Gidlewski, & Deliberto, 2014).

Wild pigs can be considered as ecosystem engineers due to the changes they catalyze on a landscape (Jones, Lawton, & Shachak, 1997). Pigs alter the composition and structure of plant communities by reducing plant survival through rooting behaviors, wallowing, and trampling (Hone, 2002; Taylor, 1993). Further, they can disperse the seeds of invasive weeds via excretion after consumption (Lynes & Campbell, 2000). Diet analysis shows that pigs will eat almost any organic substance (Schley & Roper, 2003) including plants, birds, amphibians, and other mammals. Managing wild pigs is important given their influence on actively protected areas, such as wildlife refuges, national forests, and parks, through their rapid consumption of flora and fauna (Campbell & Long, 2009; Hess, Jeffrey, Pratt, & Ball, 2010; Singer, Otto, Tipton, & Hable, 1981). Other impacts to ecosystems that are caused by wild pigs include pathogen shedding into water sources (Hampton, Spencer, Elliot, & Thompson, 2006; Jay et al., 2007), pathogen spillover (Wu et al., 2012), and viral reassortment (Hall et al., 2008; Kida et al., 1994).

Wild pigs have become a destructive and dangerous invasive species, and significant financial resources are being expended for control efforts (Bevins et al., 2014). Actions to reduce wild pig populations are ongoing throughout the United States. Despite control efforts in many states, wild pig populations continue to grow. Challenges to eradication efforts include immigration of pigs from surrounding areas, movement by humans, difficulty in detecting and removing the last few individuals, and the high fecundity of wild pigs. Wild pig populations must be reduced to zero for successful control because a few remaining individuals can reproduce leading to rapid repopulation (Barrett & Pine, 1980; Choquenot, Mcllroy, & Korn, 1996). Application of environmental DNA (eDNA) detection techniques allows for surveillance and management of invasive species that are difficult to monitor or detect by direct observation (Ficetola, Miaud, Pompanon, & Taberlet, 2008; Jerde, Mahon, Chadderton, & Lodge, 2011; Piaggio et al., 2014; Tréguier et al., 2014). Detection of invasive species using eDNA is likely to be more efficient than observational monitoring after an intensive eradication program or in the initial stages of an invasion because the probability of visually detecting a few remaining individuals is likely very low (Jerde et al., 2011; Pilliod, Goldberg, Laramie, & Waits, 2013).

Environmental DNA is DNA that is shed from an organism into the environment and can be detected in cellular or extracellular forms (Darling & Mahon, 2011; Jerde et al., 2011). Sources of eDNA include mucus, saliva, feces, urine, gametes, and shed skin or hair (Ficetola et al., 2008; Taberlet, Coissac, Hajibabaei, & Rieseberg, 2012). Environmental samples vary in the amount of DNA present due to many factors: the relative volume of sample to target DNA, size of the organism, and the volume or intensity of secretion or shedding (Ficetola et al., 2008; Klymus, Richter, Chapman, & Paukert, 2015). Depending on conditions, DNA may persist for various lengths of time in the environment (Barnes et al., 2014; Dejean et al., 2011; Ficetola et al., 2008). Conditions that are likely to affect degradation of eDNA include exposure to UVB radiation, pH, heat, and endo‐ and exonucleases in the aquatic environment (Ficetola et al., 2008; Pilliod, Goldberg, Arkle, & Waits, 2014). Another influence on DNA in the environment is microorganisms that digest and break down DNA (Dejean et al., 2011). Other challenges associated with eDNA detection include the presence of inhibitors and the sensitivity and specificity of laboratory assays. Inhibitors are humic substances that may be co‐extracted with eDNA and inhibit the performance of conventional PCR or quantitative PCR (Albers, Jensen, Bælum, & Jacobsen, 2013; Matheson, Gurney, Esau, & Lehto, 2010; Tsai & Olson, 1992) such that the eDNA detection assay does not perform as expected (McKee, Spear, & Pierson, 2015).

The design and implementation of eDNA detection methods for invasive species monitoring must be rigorously controlled through good laboratory practices and the development of assays with high sensitivity and specificity to prevent errors in detection (Goldberg et al., 2016). Despite the fact that DNA begins to degrade as soon as it is shed, and is typically found in low concentrations in the environment, eDNA detection has been an effective tool for identification of recently introduced aquatic and semiaquatic invasive species (e.g., Ficetola et al., 2008; Darling & Mahon, 2011; Piaggio et al., 2014). Application of eDNA has largely been restricted to aquatic species, limiting conservation and management efforts with this method. The concept of using eDNA in water sources to detect terrestrial wildlife has been tested with *Canis latrans* (Rodgers & Mock, 2015) and metabarcoding for terrestrial mammalian eDNA (Ushio et al., 2016) but not yet optimized in terms of detection and degradation thresholds.

Environmental DNA techniques could provide an ideal approach for detection and monitoring of wild pigs. Pigs spend time daily drinking or wallowing in water bodies (Jay et al., 2007; Taylor, 1993) for thermoregulation and protection from insects and parasites (Campbell & Long, 2009; Graves, 1984; Heinken, Schmidt, Oheimb, Kriebitzsch, & Ellenberg, 2006). Through drinking behaviors, saliva containing cells with DNA are shed into the water (Rodgers & Mock, 2015) while wallowing behaviors can lead to shedding of epithelial cells; urine and feces can also be a source of eDNA shed into the environment (Beja‐Pereira, Oliveira, Alves, Schwartz, & Luikart, 2009; Valiere & Taberlet, 2000).

Here, we test the sensitivity of an eDNA assay we developed for the detection of wild pigs (Williams, Huyvaert, Vercauteren, & Piaggio, 2017). A goal was to determine how long a pig must have contact with a water source or what behaviors are required (i.e., drinking/contact with snout verses wallowing/whole body contact) to shed sufficient DNA in water for reliable detection. Another goal was to develop an understanding of how long pig eDNA can persist in water, providing insight into how recently a pig visited the water source. An understanding of pig eDNA persistence in water could also be useful in surveillance of areas of new invasion by providing a time frame of when wild pigs were likely last in the area. Through a series of careful experiments, we are one step closer to implementing eDNA monitoring in the field for detecting invasion or monitoring success in an eradication effort.

## 2 METHODS

Laboratory work was performed at the USDA‐APHIS National Wildlife Research Center in Fort Collins, Colorado, USA. Extractions were performed in a laboratory where only low‐quantity/quality DNA was processed. All PCR and post‐PCR procedures were completed in separate rooms. Equipment, benchtops, pipettors, and fume hoods were cleaned with 10% bleach before and after any procedure.

### 2.1 Study sites

To develop an understanding of the behavior of eDNA shed by wild pigs into the environment, we built artificial wallows to mimic conditions in nature. Artificial wallows were constructed by placing a large (1,135 L) tub flush to the ground in each enclosure (group: 26 June 2014; single pig: 9 July 2014). Tubs had never been exposed to pigs prior to use. Cinder blocks were added to make the wallows shallow and accessible for pigs to enter and leave with minimal effort. We filled the water tubs with the cinder blocks to a final volume of approximately 800 L each (group: 757 L, single pig: 852 L). The pigs used for this experiment were held in captivity (0.125‐acre pen) at the USDA‐APHIS/Colorado State University Wildlife Research Facility with all necessary IACUC reviews and approvals (IACUC Protocol #13‐4638A, Colorado State University). We had two enclosures: one with 13 pigs (hereafter, “Group”) and another with a single male pig (“Single pig”).

Small waterers were set up at the USDA NWRC Mississippi Field Station in Starkville, Mississippi, to determine minimal contact and duration of contact for detection from a single pig. An automated waterer (Little Giant, Miller Manufacturing, Glencoe, MN) was used consisting of a large tank outfitted with a bowl at its base; the bowl was filled with 1.48 L of water and was refilled by pressure from the animal on a metal paddle situated on the back of the bowl. This waterer had never been used and was placed in a pen with a single pig.

### 2.2 Assay sensitivity

We used the “Single pig” wallow to determine the minimum amount of eDNA needed to accumulate in the wallow, without minimizing body contact, for a positive eDNA detection. Three 60 ml samples were collected in Nalgene bottles from the tub immediately after filling, serving as time zero samples (negative control). From then on, the pig was free to interact with the water in the wallow at will. Every 15 min for 2 hr, three 60 ml water samples were collected from the tub. Pig behavior and interactions with the water in the wallow were recorded (Appendices S1 and S2). Effort was taken to minimize the effects of human presence on pig behavior. In between sampling periods, the sampler left the pen and retreated to an area away from the pig. Samples were stored in a cooler during the sampling period, then transferred to a −80°C freezer until processing.

To test the sensitivity of the assay to detect eDNA with minimal pig interaction with the water source, we limited the pig to snout/mouth contact only with the galvanized steel waterers. Three 60 ml samples were collected in Nalgene bottles at time zero and every 15 min for 2 hr. The pig was allowed to drink from the waterer at will and, after samples were collected, the waterers automatically refilled to capacity. Pig behavior with the waterer was documented (Appendices S1 and S2). Samples were shipped from the field station to the laboratory (NWRC, Fort Collins, CO, USA) on dry ice and then stored in a −80° freezer until processing.

### 2.3 Persistence

Artificial wallows were left in both the single pig and group pens for 1 week after installation in which the pigs actively used the water source. The wallows were turbid from soil introduced by the pigs. After 1 week of use, eighty‐seven 60 ml samples were collected in Nalgene bottles from each of the wallows. A table was set up in an enclosed building with open sides allowing for temperature fluctuation and some exposure to UV from sunlight; the 87 samples were placed on the table to allow for environmental degradation of the eDNA over time. Three of the 87 samples were taken at the start of the time series to serve as time zero of degradation for controls, then three samples were collected from the table every 12 hr over the next 2 weeks to measure eDNA degradation over time. All samples were carried in a cooler from the outdoor enclosure into the laboratory (~5‐min walk) and stored in a −80°C freezer until processing.

### 2.4 eDNA capture

All samples were processed using our optimized eDNA capture and qPCR protocols previously developed for wild pig eDNA detection (Williams et al., 2017). Water samples (15 ml) were centrifuged at 9,000 g for 15 min, and DNA was extracted from the pellet with the DNeasy mericon Food Kit (Qiagen, Hilden, Germany). Extracts were run through inhibitor removal treatment columns (Zymo Research, Irvine, CA, USA). Primers were used from the Williams et al., 2017 study targeting a fragment in the D‐loop region of *Sus scrofa* (NC00845, BLAST). Each qPCR reaction was a 30 μl reaction containing 15 μl Taqman environmental mastermix (Life Technology), 1 μl of each primer (10 μmol/L), 1 μl of the probe (2.5 μmol/L), 1 μl BSA, 6 μl distilled water, and 5 μl of DNA extract run on a Biorad real‐time PCR thermocycler (Biorad, Hercules, CA, USA). The real‐time thermocycling program involved 10 min at 95°C, 50 cycles of 95°C for 15 s, and 1 min at 52°C, and we included a standard curve with each run using dilutions of a synthetic sequence of our target amplicon (gBlocks^®^ Gene Fragments, IDT). We included a negative control in each set of extractions to monitor for contamination.

Each qPCR set included a “no template” negative control including only PCR reagents to monitor for contamination. Each extracted water sample was run in triplicate via qPCR. We considered a positive in any qPCR replicate as a detection; a replicate was considered positive if the measured DNA concentration was above our LOD (1 copy/μl). We assumed no false positives. All qPCR runs included a standard curve and met performance requirements (*E* = 90%–110%, *R* ^2^ > .99, Slope = −3.1 to −3.6).

### 2.5 Statistical analysis

#### 2.5.1 Assay sensitivity

We did not apply a statistical test for detection probabilities for the “Single pig” and waterer detection experiments; we simply recorded the number of successful samples per time point because all qPCR replicates were positive after time zero. We analyzed the accumulation of eDNA over 2 hr in naïve water using robust regression including sample as a random effect (J. Hoeting, Personal Communication, February 25, 2016), allowing for inclusion of outliers that we felt were of biological importance due to events such as DNA clumping at the field site or throughout the extraction and amplification processes. We compared the DNA concentrations pre‐ and postpig contact with water with the prepig water samples serving as a negative control. We included all measured DNA concentrations that resulted in a positive time point sample in this analysis.

#### 2.5.2 Persistence

For the persistence experiment, we used a generalized linear mixed model with a random effect of individual samples per time point to determine whether the hours of degradation affected our probability of detecting eDNA. This was performed on the proportion of qPCRs that were positive (total qPCRs = 9 per time point) for each of the 12‐hr collection time points. We then combined the degradation data from the “Single pig” and “Group” and evaluated a set of logistic regression models. The a priori candidate set of models included the effects of hours of degradation, whether or not the eDNA was shed from a “Group” of pigs or the “Single pig,” and the interaction of group and hours on detection of eDNA. Models were ranked by AICc values where the model with the lowest AICc was considered the best‐supported model given the data and model set.

We used the highest ranking model (Detection ~ Hour + Group + Group*Hour) to develop a predictive model of the amount of time necessary for previously shed eDNA to degrade, assuming no new introduction of pig DNA occurred. We also modeled the decline in the concentration of eDNA in water over time to see how degradation time affects eDNA. For this analysis, we used a robust regression to account for the outliers that we thought were of biological significance potentially due to DNA clumping during the sampling or extraction and amplification process. Statistical analyses were conducted in R x64 3.1.2.

## 3 RESULTS

### 3.1 Assay sensitivity

We amplified the 101‐bp fragment of the *Sus Scrofa* D‐loop region from all water samples collected from both the “Single pig” wallow (USDA‐APHIS/Colorado State University Wildlife Research Facility) and the waterers used by a single pig (Mississippi field station), where we detected 3/3 positive qPCR replicates for each of three samples collected for each time point from 15 min to 2 hr.

### 3.2 eDNA accumulation

**Figure 1:**
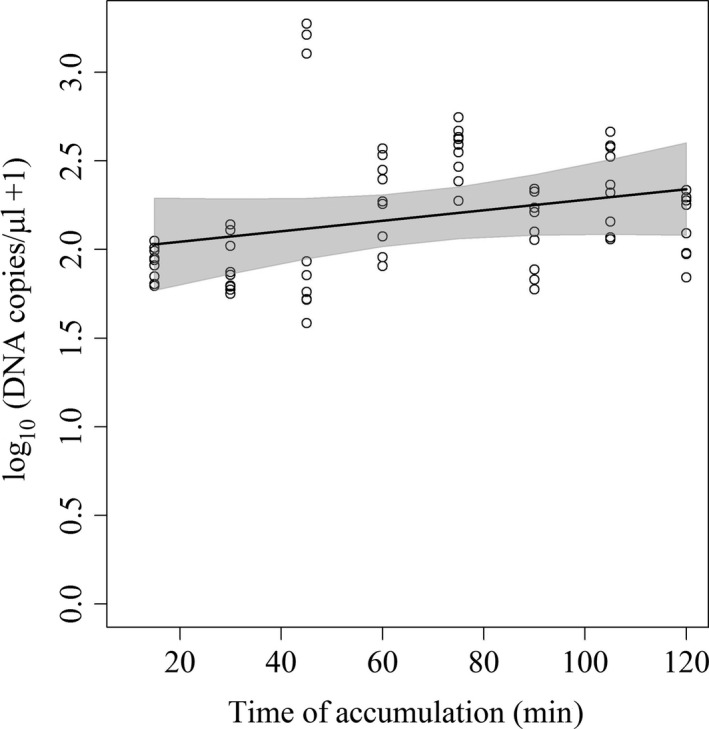
Robust regression of log‐transformed DNA copies/μl in response to minutes of eDNA accumulation through the single pig contact with wallow water

Time had a statistically significant effect on the concentration of eDNA for the “Single pig” wallow trial (*p* = .044 (Figure 1). The concentration of eDNA measured increased as the time of accumulation increased (β = 0.00355, *SE* = 0.001768).

### 3.3 Persistence

**Figure 2:**
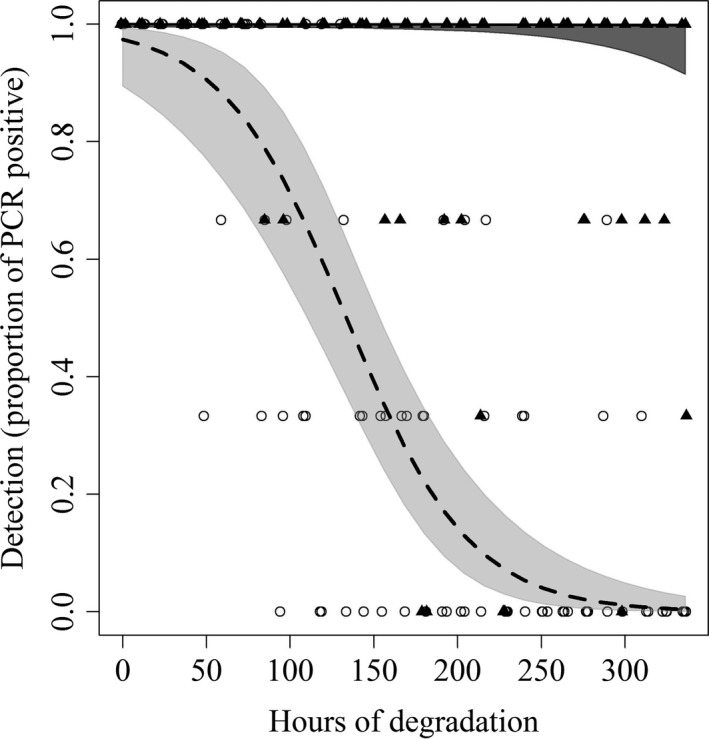
Binomial regression of detection of eDNA (proportion of qPCRs positive) over the course of a 2‐week period during which eDNA was allowed to degrade

There was a statistically significant effect of time on the detectability of degrading DNA for the “Single pig” trial (*p* < .0001) but not for the “Group” trial (*p* = .233) (Figure 2). The best‐supported model of the combined analysis was the model incorporating the interaction of group size (single pig or group) and hours of degradation (Table 1).

**Table 1:** Candidate models for the probability of detecting degrading eDNA over time ranked by AICc. A generalized linear model (glm) was used to predict persistence. The best‐supported model includes all variables and an interaction between group and hours of degradation. The AICc values, number of parameters (*K*) in each model, and the log likelihood are reported for each candidate model

Model	AICc	Δ AICc	*K*	LL
Detection ~ Hour + Group + Group*Hour	275.18	0.00	5	−132.41
Detection ~ Hour + Group	287.18	11.99	4	−139.47
Detection ~ Group	344.14	68.95	3	−169.00
Detection ~ Hour	503.22	228.04	3	−248.54

**Figure 3:**
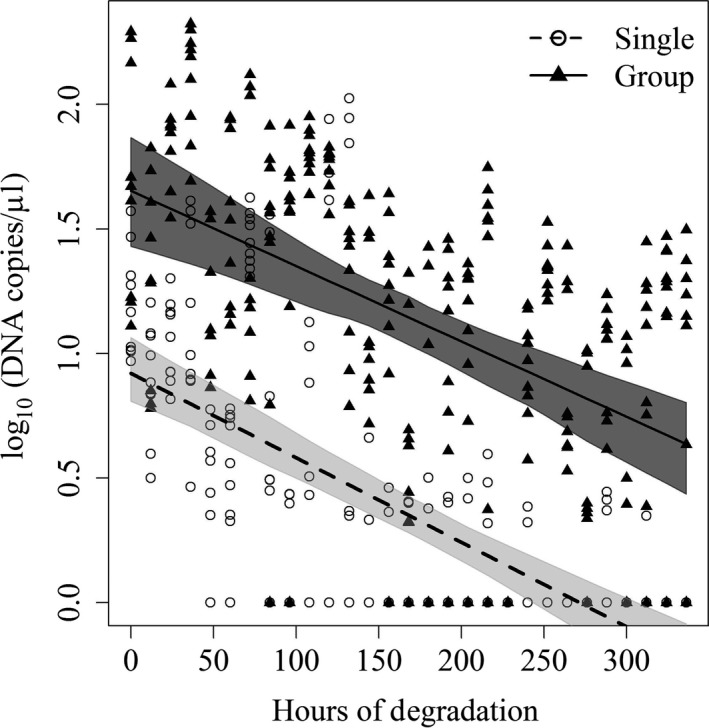
Robust regression of log‐transformed DNA concentration over time showing the effect of degradation time on the amount of eDNA detected in water samples

We used a generalized linear mixed model to show the effect of time on the combined single pig and group data (Figure 3). In the field, it will be unknown if a single pig or group of pigs last interacted with the water body; therefore, we were interested in looking at the degradation rate without being able to account for group size.

### 3.4 Quantification of eDNA degradation

Concentration decreased with degradation time; this effect was statistically significant (*p* < .0001) for both “Group” and for the “Single pig” (Figure 2). Hours of degradation had a negative effect on the measured DNA concentration in the water samples for the “Group” (β = −0.0030, *SE* = 0.0005) and for the “Single pig” (β = −0.0034, *SE* = 0.0004).

## 4 DISCUSSION

Detection of eDNA offers a promising tool to monitor habitats for new invaders either transported from afar or on the invasion front. We demonstrated that eDNA shed by wild pigs can be detected in water after only 15 min of exposure by a single pig. This was true even when the single pig's contact was minimal through nose/mouth contact with the water. This finding is important as hunting pressure may change pig behavior and cause them to be on the move and only interact with water sources to drink rather than wallow (Gaston, 2008; Sodeikat & Pohlmeyer, 2003).

The tests for persistence of eDNA in the wallow samples showed that the number of individuals shedding into the water can affect how long degrading eDNA can be detected (Figure 2). We did not run the persistence experiment long enough (>2 weeks) for the group eDNA to completely degrade such that we could no longer detect it.

Understanding the relationship between the size of the population of pigs (single, few, many) to how long eDNA may persist in the environment is useful when applied to management activities. Our results show that if a lone pig were removed from an environment where it was thought to be solitary, and eDNA was detected 20 days (465 hr) later, the eDNA likely came from a new invader and not the remnant eDNA from pig that had been removed through management activities (Table 2). A clear difference was observed between our “Single pig” and “Group” degradation rates, variability will be expected in field results. If this degradation trend continued according to the model, there could potentially be detectable DNA from a group of pigs on the landscape up to 72 days after removal. Persistence studies with longer duration for degradation would need to be conducted to provide reliable management recommendations on how soon to sample after elimination of more than a single pig. Our pen experiments could be considered to have a higher density of pigs than what would be expected on the landscape due to facility limitations (0.125‐acre enclosures resulting in a high density of pigs) and perhaps contribute to a higher DNA load in our water system compared to a wild setting. Granted, the number of pigs shedding into the system is only one factor affecting persistence and both biotic (pig behavior, gender, body mass) and abiotic (UV, temperature) also influenced our results though were not directly tested here. Although the samples were treated for inhibitor removal, inhibitors that were not successfully removed after the treatment could still affect detection.

**Table 2:** Parameter estimates from the best‐supported model of the probability of detecting degraded eDNA over time (“hour”). Samples were collected from pig wallows with only a single pig or a group of pigs (“group”). Estimates were derived from the best‐supported model: Detection ~ Hour + Group + Group*Hour

Coefficients	Estimate	*p* Value	*SE*
Intercept	4.232	<.0001	0.826
Hour	−0.031	<.0001	0.005
Group	1.342	.153	0.938
Group*Hour	0.019	.0004	0.005

Emphasis should be placed on changes in eDNA concentration over time due to degradation rather than detection alone. Although we observed positive qPCR detections in the later time points, the DNA concentrations of the samples decreased throughout the duration of the degradation trial. If this method were applied in a management setting, samples collected regularly after an eradication attempt could provide useful information on whether or not new invasions by pigs were occurring. This method would require continuous sampling but may not require intensive field work if samples were collected monthly for monitoring an area in question.

In both the detection and persistence experiments, DNA concentrations at each time point showed a great deal of variation. Detection of environmental DNA is inherently stochastic, in that the eDNA is not distributed homogeneously in the environment and the probability of detection varies. Recently, Furlan, Gleeson, Hardy, and Duncan (2015) found that eDNA detection was dependent upon the concentration, dispersion, and survey method. They found that DNA was not dispersed evenly in water samples, rather it was spatially clumped throughout the site causing some samples to contain few or no DNA copies where DNA was in fact shed into the system. We observed similar between‐sample variation, although we did not assess whether it was due to clumping in the wallow or whether it occurred during the extraction process. The outliers in the eDNA accumulation graph (Figure 1) could be attributed to this phenomenon. To address this issue, recent studies have applied occupancy approaches to estimate the probability of detection of eDNA and factors contributing to uncertain detection (Hunter et al., 2015; Pilliod, Goldberg, Arkle, & Waits, 2013; Schmelzle & Kinziger, 2016). Occupancy approaches take into account detection uncertainty, whether it is due to eDNA clumping or heterogeneity of capture efficiency, allowing for estimates of occupancy relative to varying detection methods or other sources of heterogeneity (Furlan et al., 2015; MacKenzie et al., 2006; Schmidt, Kéry, Ursenbacher, Hyman, & Collins, 2013).

## 5 CONCLUSION

We demonstrated that an eDNA assay can be sensitive enough to detect low quantities of eDNA shed by a terrestrial mammal that has limited interaction with water. We also provide estimates of how long pig eDNA can persist in turbid water environments depending on whether it is shed from a single pig or a group of pigs. With appropriate field optimization, our method will be useful for detecting new invaders and determining efficiency of eradication efforts.

## CONFLICT OF INTEREST

None declared.

## AUTHOR CONTRIBUTIONS

K.E.W. and A.J.P. conceived the concept of the study. K.E.W., A.J.P., K.P.H., and K.C.V. participated in study design. K.E.W. collected water samples and conducted laboratory work. A.J.D., K.E.W., and K.P.H. performed data analysis. K.E.W. prepared the manuscript with help from A.J.P., K.P.H., A.J.D., and K.C.V. All authors read and approved the final manuscript.
