# Supplementary material for: Detection and persistence of environmental DNA from an invasive, terrestrial mammal
Source: Ecol Evol. 2017 Dec 3;8(1):688–95. doi: 10.1002/ece3.3698 (PMC5756866; doi:10.1002/ece3.3698)
Supplement: Supplementary file 1 [file ECE3-8-688-s001.docx]

Supporting Information

These data are a record of observations during the eDNA accumulation experiment involving a single pig interacting with a wallow and a single pig interacting with a waterer.

Key:

* = Sample collected

Triplicates of each sample in 60 ml bottles

**Single Pig Wallow Sampling**

July 9, 2014

Filled tub at 7:15AM

94.6 L at a time

~852 L

71.8°F at 8:10AM

8:10 AM *

Time zero sample collected

8:10AM Pig in pen

8:19 AM walked through tub

8:21 AM walked through tub

8:25 AM *

8:30 AM

Stood in water/rooting under water

8:40 AM *

No movement in tub

8:55 AM *

8:57 AM

Drank/walked in tub

9:03 AM

Drank/walked through tub

9:06 AM

Drank/walked in tub

9:10 AM *

9:12 AM

Pig in tub

9:15 AM

Pig in tub

9:17 AM

Walked through tub

9:19 AM

Walked through tub

9:25 AM *

9:29 AM

Pig in tub

9:40 AM *

9:45 AM

Laid in tub

9:55 AM *

10:00 AM

Wallowed

10:10 AM *

**Waterer Sampling**

Mississippi Field Station

8:53 AM*

8:57 AM

Pig in pen

Pig drank for a few seconds

9:04 AM

Pig drank

9:07 AM

Pig drank

9:12 AM

Pig drank immediately before sample collection

9:13 AM *

9:22 AM

Pig drank for a few seconds

9:27 AM *

9:42 AM *

9:43 AM

Pig drank for 3-4 seconds

9:57 AM*

9:58 AM

Stuck nose in for a second

10:12 AM*

10:27 AM*

10:30 AM

Pig stuck nose in for a second

10:42 AM*

10:57 AM*
